# Treatment of severe COVID-19 with convalescent plasma in Bronx, NYC

**DOI:** 10.1172/jci.insight.142270

**Published:** 2021-02-22

**Authors:** Hyun ah Yoon, Rachel Bartash, Inessa Gendlina, Johanna Rivera, Antonio Nakouzi, Robert H. Bortz, Ariel S. Wirchnianski, Monika Paroder, Karen Fehn, Leana Serrano-Rahman, Rachelle Babb, Uzma N. Sarwar, Denise Haslwanter, Ethan Laudermilch, Catalina Florez, M. Eugenia Dieterle, Rohit K. Jangra, J. Maximilian Fels, Karen Tong, Margarette C. Mariano, Olivia Vergnolle, George I. Georgiev, Natalia G. Herrera, Ryan J. Malonis, Jose A. Quiroz, Nicholas C. Morano, Gregory J. Krause, Joseph M. Sweeney, Kelsie Cowman, Stephanie Allen, Jayabhargav Annam, Ariella Applebaum, Daniel Barboto, Ahmed Khokhar, Brianna J. Lally, Audrey Lee, Max Lee, Avinash Malaviya, Reise Sample, Xiuyi A. Yang, Yang Li, Rafael Ruiz, Raja Thota, Jason Barnhill, Doctor Y. Goldstein, Joan Uehlinger, Scott J. Garforth, Steven C. Almo, Jonathan R. Lai, Morayma Reyes Gil, Amy S. Fox, Kartik Chandran, Tao Wang, Johanna P. Daily, Liise-anne Pirofski

**Affiliations:** 1 Division of Infectious Diseases, Department of Medicine, Albert Einstein College of Medicine and Montefiore Medical Center, Bronx, New York, USA.; 2 Department of Microbiology and Immunology and; 3 Department of Biochemistry, Albert Einstein College of Medicine, Bronx, New York, USA.; 4 Department of Pathology and; 5 Department of Oncology, Albert Einstein College of Medicine and Montefiore Medical Center, Bronx, New York, USA.; 6 Department of Chemistry and Life Science, United States Military Academy at West Point, West Point, New York, USA.; 7 Department of Developmental & Molecular Biology,; 8 Institute for Aging Research, and; 9 Department of Physiology and Biophysics, Albert Einstein College of Medicine, Bronx, New York, USA.; 10 Albert Einstein College of Medicine, Bronx, New York, USA.; 11 Department of Epidemiology and Population Health, Albert Einstein College of Medicine, Bronx, New York, USA.; 12 Network Performance Group, Montefiore Medical Center, Bronx, New York, USA.; 13 Division of Hospital Medicine, Department of Medicine, Albert Einstein College of Medicine and Montefiore Medical Center, Bronx, New York, USA.

**Keywords:** COVID-19, Infectious disease, Immunoglobulins

## Abstract

Convalescent plasma with severe acute respiratory disease coronavirus 2 (SARS-CoV-2) antibodies (CCP) may hold promise as a treatment for coronavirus disease 2019 (COVID-19). We compared the mortality and clinical outcome of patients with COVID-19 who received 200 mL of CCP with a spike protein IgG titer ≥ 1:2430 (median 1:47,385) within 72 hours of admission with propensity score–matched controls cared for at a medical center in the Bronx, between April 13 and May 4, 2020. Matching criteria for controls were age, sex, body mass index, race, ethnicity, comorbidities, week of admission, oxygen requirement, D-dimer, lymphocyte counts, corticosteroid use, and anticoagulation use. There was no difference in mortality or oxygenation between CCP recipients and controls at day 28. When stratified by age, compared with matched controls, CCP recipients less than 65 years had 4-fold lower risk of mortality and 4-fold lower risk of deterioration in oxygenation or mortality at day 28. For CCP recipients, pretransfusion spike protein IgG, IgM, and IgA titers were associated with mortality at day 28 in univariate analyses. No adverse effects of CCP were observed. Our results suggest CCP may be beneficial for hospitalized patients less than 65 years, but data from controlled trials are needed to validate this finding and establish the effect of aging on CCP efficacy.

## Graphical abstract

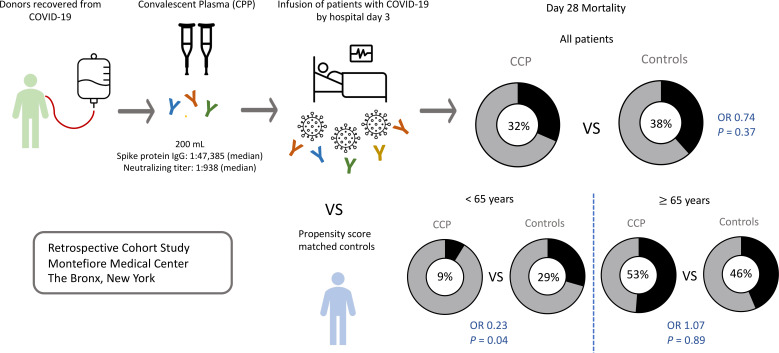

## Introduction

Severe acute respiratory syndrome coronavirus 2 (SARS-CoV-2) (1), a highly transmissible enveloped positive-strand RNA virus (2), is the causative agent of the coronavirus disease 2019 (COVID-19) pandemic (3). The first case was reported in December 2019, and by November 2020 more than 61 million infections were reported worldwide, with one fifth of the cases and deaths occurring in the United States (4). In April 2020, New York City (NYC), especially the borough of the Bronx, was an early epicenter of the COVID-19 pandemic in the United States (5, 6). Since then, an antiviral that reduced duration of illness (7), remdesivir, received FDA approval on October 22, 2020, and corticosteroids, which reduced mortality in severely ill patients in a large randomized clinical trial and prospective meta-analysis (8, 9), have become standard of care. As of November 2020, there is no approved therapy for COVID-19 that reduces mortality of hospitalized patients with respiratory manifestations of severe or life-threatening disease.

Convalescent plasma (CP) obtained from recovered persons was deployed for previous respiratory pandemics, including 1918 and 2009 influenza and SARS (10–13). Given the lack of established therapies for COVID-19, CP containing SARS-CoV-2 antibodies (CCP) was proposed as a therapeutic option early in the pandemic (14). As of November 2020, it has shown a possible benefit in multiple studies. In early case series, CCP-treated patients exhibited viral clearance and reductions in inflammatory markers (15–19). Observational studies comparing CCP-treated patients to retrospective controls showed a reduction in mortality in nonintubated patients and/or those transfused within 72 hours of hospitalization with high-titer CCP (20–24). Analysis of a subset of more than 3000 CCP recipients in an open-label study found a dose response whereby nonintubated patients who received high-titer CCP had lower mortality than those who received low-titer CCP (22). Several randomized controlled trials (RCTs) have not shown a benefit of CCP but were limited by premature termination due to a lack of patients to recruit (25, 26). One trial found CCP had an antiviral effect, but there was no effect on mortality (27); another found a reduction in mortality, albeit with a very small sample size (28); and another was terminated due to the presence of neutralizing antibodies in CCP recipients at the time of transfusion, despite being on track to meet the primary endpoint (29). A recent double-blind, placebo-controlled, multicenter RCT did not show an effect of CCP on mortality (30). Evidence of safety and possible benefit led the FDA to issue an emergency use authorization for CCP use in hospitalized patients with COVID-19 on August 23, 2020.

We treated 103 patients at Montefiore Medical Center (MMC), a 1491-bed tertiary medical center in the Bronx, New York, with serious or life-threatening COVID-19 with CCP between April 13 and May 4, 2020, and conducted a propensity score–matched study. Herein, we report mortality and clinical and laboratory findings of CCP recipients compared with matched controls.

## Results

### Baseline characteristics of CCP recipients and retrospective controls.

**Figure 1:**
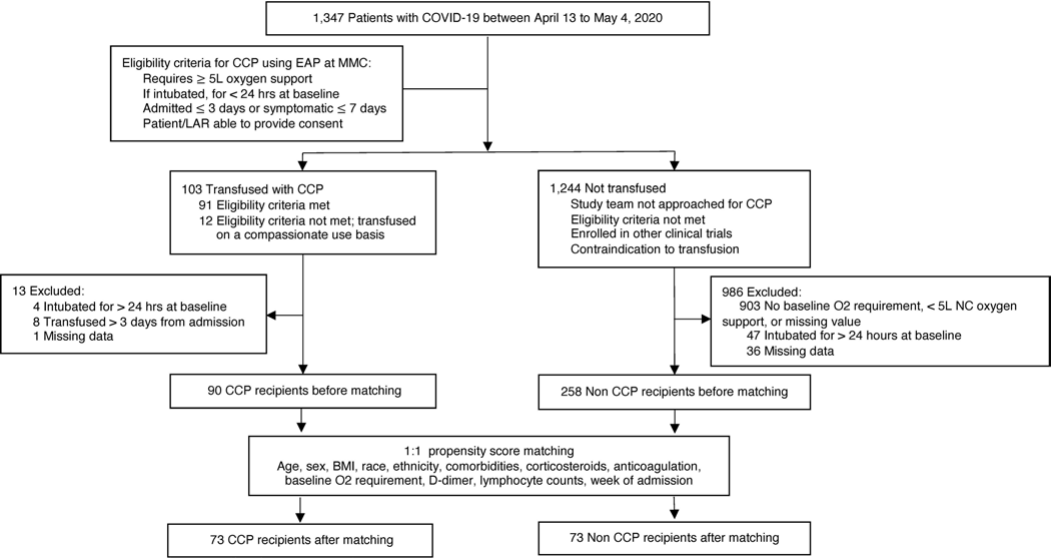
Enrollment of study patients and distribution of study cohorts. Study baseline was defined as time of CCP transfusion for CCP recipients and admission day 2 for non-CCP recipients. COVID-19, coronavirus disease 2019; CCP, COVID-19 convalescent plasma; EAP, expanded access protocol; LAR, legally authorized representative; MMC, Montefiore Medical Center; NC, nasal cannula.

One hundred three (*n* = 103) patients were enrolled in the Mayo Clinic expanded access protocol (EAP) (31) and treated with one 200 mL unit of CCP within 72 hours of hospital admission. Clinical status and mortality on day 28 posttransfusion was compared to retrospective propensity score–matched controls identified by querying the electronic medical record (Figure 1). Analysis included 90 CCP recipients and 258 controls after exclusion of 12 patients who did not meet the eligibility criteria because of mechanical ventilation for more than 24 hours or CCP transfusion more than 72 hours after admission and 1 patient with missing data (Figure 1). Compared with controls, CCP recipients were younger (median 66 vs. 72 years, *P* = 0.002), had higher BMI (28 vs. 27 kg/m^2^, *P* = 0.05), and had lower rates of congestive heart failure (18 vs. 29%, *P* = 0.03) and chronic kidney disease (29 vs. 41%, *P* = 0.03). At baseline, a lower proportion of CCP recipients were on low-flow oxygen support (68 vs. 81%, *P* < 0.0001), and a higher proportion required mechanical ventilation (20 vs. 6%, *P* < 0.0001), had lower baseline lymphocyte counts (0.8 vs. 1.0 × 10^9^/L, *P* = 0.001), and received systemic corticosteroids (93 vs. 63%, *P* < 0.0001) (Supplemental Table 1; supplemental material available online with this article; https://doi.org/10.1172/jci.insight.142270DS1). After propensity score matching, 73 CCP recipients and 73 control patients were well balanced for all matching variables except in a subgroup analysis stratified by age; CCP recipients at least 65 years had lower lymphocyte counts than controls (median, 0.8 vs. 1.0 × 10^9^/L) (Table 1 and Supplemental Figure 1).

### CCP SARS-CoV-2 spike protein and neutralizing antibody titers.

Of the 200 mL units of CCP administered in this study, 95 of 103 were obtained from 46 persons who donated CCP at MMC in April 2020. SARS-CoV-2 spike protein IgG endpoint titers were measured in CCP obtained from these donors using the MMC in-house research full-length spike protein ELISA (32). Median IgG, IgM, and IgA titers were, respectively, 1:47,385 (IQR, 21,870–65,610; *n* = 46), 1:810 (IQR, 810–2430; *n* = 43), and 1:90 (IQR, 90–270; *n* = 43). Median neutralizing antibody (NAb) titer by pseudovirus neutralization assay was 1:938 (IQR, 407–2784; *n* = 42). There was a direct correlation between NAb titer and spike protein IgG (Spearman *r* = 0.78, *P* < 0.0001) and IgM (*r* = 0.58, *P* < 0.0001) and weak correlation with IgA (*r* = 0.29, *P* = 0.05) titers (Supplemental Figure 2). For the 8 patients who did not receive MMC donor CCP, 7 received units that tested as “reactive” by the New York State Department of Health Wadsworth Center’s SARS-CoV-2 Microsphere Immunoassay for anti-nucleocapsid antibody detection (33), and 1 received CCP with a spike protein IgG titer of 1:320 measured by the in-house spike protein ELISA at Mount Sinai Hospital (34).

### Comparison of clinical outcomes of CCP recipients and controls.

**Figure 2:**
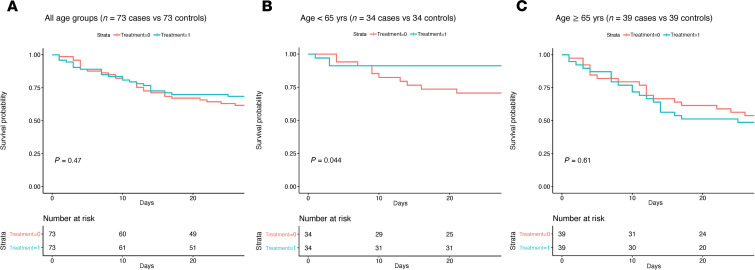
Kaplan-Meier plots of the probability of survival from time of transfusion to day 28 in CCP recipients (*n* = 73) versus matched controls (*n* = 73). (**A**) All age groups. (**B**) Age < 65 years. (**C**) Age ≥ 65 years. The *P* value of a log-rank test is shown for each plot.

There was no difference in mortality between 73 CCP recipients and 73 propensity score–matched controls by day 28 (*P* = 0.47, Kaplan-Meier log-rank test) (Figure 2). To account for the potential interaction between age and CCP treatment (*P* = 0.11, interaction term), analysis was stratified by age. Compared with matched controls, CCP recipients younger than 65 years had lower mortality by day 28 (*P* = 0.04, Kaplan-Meier log-rank test), whereas the mortality of CCP recipients and matched controls at least 65 years did not differ significantly (*P* = 0.61, Kaplan-Meier log-rank test) (Figure 2). There was no difference in mortality between groups when CCP recipients and controls were stratified by baseline oxygen requirement (Supplemental Figure 3).

**Figure 3:**
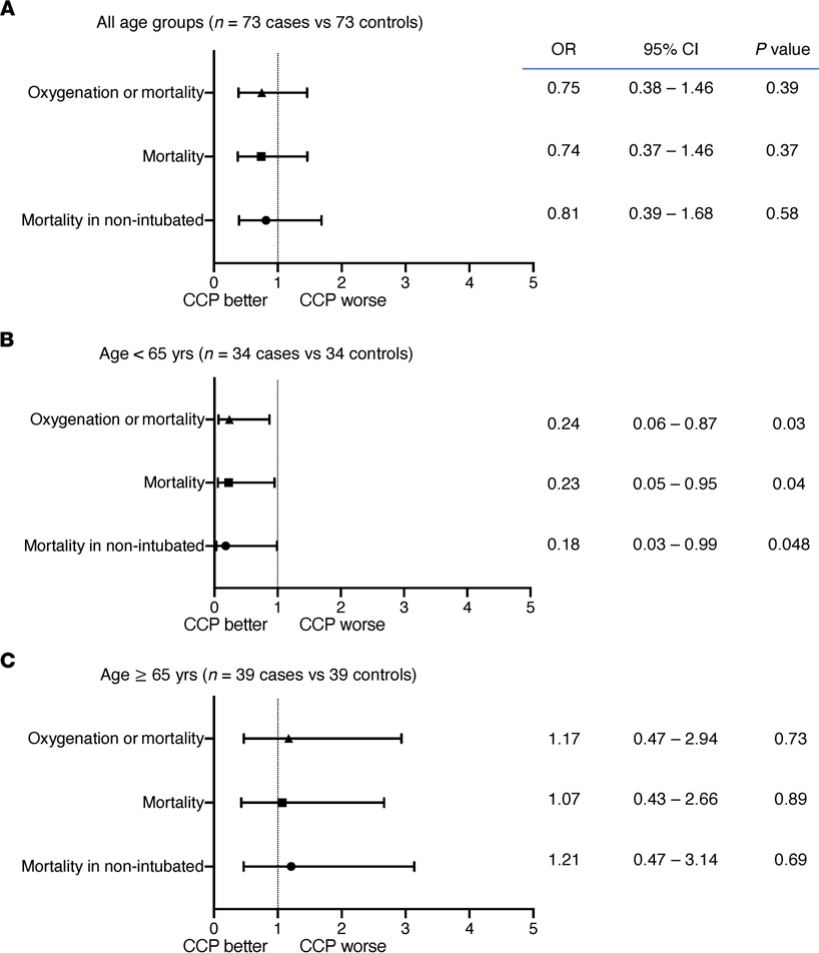
Day 28 outcomes for CCP recipients (*n* = 73) versus matched controls (*n* = 73) presented by OR and 95% confidence intervals using a logistic regression model. (**A**) All age groups (*n* = 73 cases vs. 73 controls). (**B**) Age < 65 years (*n* = 34 vs. 34). (**C**) Age ≥ 65 years (*n* = 39 vs. 39).

There was no significant difference between CCP recipients and matched controls in all-cause mortality at 28 days (31.5 vs. 38.4%; OR, 0.74; 95% CI, 0.37–1.46; *P* = 0.37) (Figure 3). When stratified by age, CCP recipients younger than 65 years had a 4-fold decrease in risk of mortality (8.8 vs. 29.4%; OR, 0.23; 95% CI, 0.05–0.95; *P* = 0.04) and a 4-fold decrease in risk of deterioration in oxygenation or mortality (11.8 vs. 35.3%; OR, 0.24; 95% CI, 0.06–0.87; *P* = 0.03). There was no significant difference in mortality of CCP recipients 65 years or older (52.6 vs. 45.9%; OR, 1.07; *P* = 0.89) (Figure 3 and Table 1). Among the 103 CCP recipients, mortality at day 28 was associated with time from symptom onset to transfusion (OR, 1.12; 95% CI, 1.01–1.24; *P* = 0.04), earlier week of admission (OR, 2.22; 95% CI, 1.00–5.00; *P* = 0.05), and being Hispanic/Latinx in ethnicity compared with not being Hispanic/Latinx in ethnicity (OR, 8.33; 95% CI, 1.69–33.3; *P* = 0.009), adjusted for age, sex, BMI, race, ethnicity, comorbid conditions, week of admission, duration of symptoms, baseline oxygen requirement, corticosteroids, anticoagulation use, D-dimer, and lymphocyte counts (Table 2). There was no significant association between CCP NAb or spike protein IgG titers and mortality or oxygenation status in CCP recipients.

Multivariable analysis of 90 CCP recipients and 258 controls adjusted for covariates age, sex, BMI, race, ethnicity, comorbid conditions, week of admission, baseline oxygen requirement, corticosteroids, anticoagulation use, D-dimer, and lymphocyte counts did not show any difference in outcome between the 2 groups. When stratified by age, CCP recipients younger than 65 years had lower mortality or deterioration in oxygenation, but this was not statistically significant (OR, 0.23; 95% CI, 0.04–1.19; *P* = 0.08) (Supplemental Figure 4). Additionally, multivariable analysis indicated that age, use of mechanical ventilation at baseline, use of systemic corticosteroids, not being on anticoagulation in patients ≥ 65 years, and earlier week of admission were associated with mortality at day 28 adjusted for covariates (Supplemental Table 2). Corticosteroid use was associated with mortality in patients receiving low-flow oxygen at baseline (adjusted OR, 2.68; 95% CI, 1.27–5.68; *P* = 0.009) when analysis was stratified by baseline oxygen requirement.

### CCP safety and adverse events.

There were no adverse reactions, including no instances of transfusion-related acute lung injury or transfusion-associated circulatory overload attributable to CCP administration.

### CCP recipient SARS-CoV-2 spike protein antibody titers.

**Figure 4:**
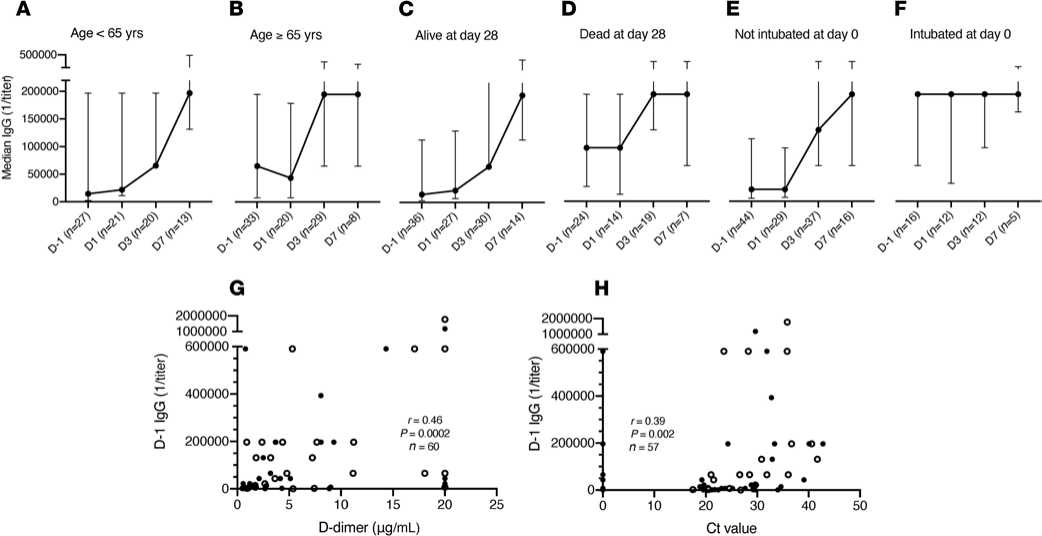
SARS-CoV-2 spike protein IgG titers determined by ELISA at baseline (day –1) and 1, 3, and 7 days after transfusion in CCP recipients. (**A**) Age < 65 years. (**B**) Age ≥ 65 years. (**C**) Alive at day 28. (**D**) Died by day 28. (**E**) Not intubated on day of transfusion. (**F**) Intubated on day of transfusion. Correlation between baseline spike protein IgG titer and (**G**) D-dimer and (**H**) cycle threshold (Ct) value from initial nasopharyngeal SARS-CoV-2 RT-PCR in CCP recipients. The median titers and IQRs are shown on the *y* axis for each time point shown on the *x* axis (**A**–**F**). The *x* axis shows days relative to CCP transfusion (**A**–**F**). Open circles show patients who died by day 28 (**G** and **H**). r, Spearman’s correlation coefficient; D, day; Ct value, cycle threshold value.

We measured CCP recipient spike protein IgG, IgM, and IgA in remnant serum samples obtained before (day –1, D –1) and after transfusion (Figure 4). Baseline spike protein IgG, IgM, and IgA were significantly higher in patients mechanically ventilated at enrollment (*P* = 0.009, *P* = 0.01, *P* = 0.02, respectively) and those who died by day 28 (*P* = 0.02, *P* = 0.02, *P* = 0.002, respectively) (Supplemental Table 3, Figure 4, and Supplemental Figure 5). There was no association between baseline antibody titers and time from symptom onset to transfusion or time from hospital admission to transfusion. Plateau in median IgG after CCP administration was reached earlier in patients ≥ 65 than < 65 years, mechanically ventilated at baseline versus not, and those who died by versus those alive at day 28 (Figure 4). Baseline IgA was higher in patients ≥ 65 than those < 65 years (*P* = 0.04) (Supplemental Table 3).

There was a direct association between mortality at day 28 and baseline (D –1) IgG (OR, 1.4; 95% CI, 1.04–1.78; *P* = 0.03), IgM (OR, 1.39; 95% CI, 1.00–1.92; *P* = 0.048), and IgA (OR, 1.45; 95% CI, 1.11–1.91; *P* = 0.007) in the univariate analyses but not in the multivariable analysis adjusted for covariates (Table 2). Baseline spike protein IgG titer was significantly correlated with D-dimer (*r* = 0.46; *P* = 0.0002; *n* = 60) (Figure 4). In addition, there was a direct, albeit weak, correlation between baseline spike protein IgG titer and detected viral load measured by Ct value of nasopharyngeal SARS-CoV-2 reverse-transcriptase PCR (RT-PCR) (*r* = 0.39; *P* = 0.002; *n* = 57) (Figure 4). There was no correlation between Ct value and age, duration of illness, or D-dimer.

### CCP recipient inflammatory and hematology measures.

There was no significant difference in change in lymphocyte counts, D-dimer, or C-reactive protein (CRP) between day 0 and 28 in CCP recipients compared to controls (data not shown).

## Discussion

CCP has been used as an investigational treatment for COVID-19 since the early days of the pandemic. Numerous observational studies report safety and signals of possible efficacy of CCP in hospitalized patients with COVID-19 (15, 20, 22–25, 35, 36). Here, we report the mortality and clinical outcomes of a cohort of 73 patients with severe to life-threatening COVID-19 who were transfused with 1 unit of CCP by 72 hours of hospitalization and 73 propensity score–matched controls. There was no significant difference in mortality or improvement in oxygenation in CCP recipients compared to controls. Although treated within 72 hours of hospitalization, CCP-treated patients had symptom duration of 5–9 days and multiple indicators of severe or life-threatening disease, including lymphopenia, elevated D-dimer levels, and the need for supplemental oxygen. In this regard, our findings are similar to those of other studies in which there was no benefit of CCP in hospitalized patients with severe COVID-19 (25, 27, 30). Nonetheless in a subset of patients stratified by age, CCP recipients younger than 65 years had significantly lower risk of mortality and deterioration in oxygenation by day 28 than controls (*P* = 0.04). Age and duration of symptoms were independently associated with mortality at day 28 in CCP recipients. No adverse reactions were directly attributable to CCP.

There was no evidence of benefit of CCP in patients 65 years or older. Age, a well-documented risk factor for COVID-19 severity and mortality (37, 38), was significantly associated with mortality in unadjusted and adjusted analyses. Patients at least 65 years had a higher frequency of comorbid conditions; higher D-dimer, CRP, and SARS-CoV-2 IgA values; and lower lymphocyte levels than those less than 65 years, all of which are markers of severe disease (39–44). Notably in published case-control studies in which CCP was associated with reduced mortality, median ages of patients were less than 60 years (21, 23, 45), and in the large open-label Mayo Clinic study in which there was a signal of reduced mortality in patients who received high-titer CCP, 44% of the cohort was <60 years, 70% of the cohort was <70 years, and CCP was less effective in those >80 years (22). In addition, a small RCT comparing 80 patients randomized to CCP versus standard of care posted on a preprint server on November 29, 2020, found a benefit of CCP in patients less than 67 years but not in the entire cohort with a median age of 61 years (46). These data along with ours suggest that aging may have a detrimental effect on CCP efficacy.

In a case-control study that also did not show a signal of CCP efficacy, Rogers et al. found a higher rate of hospital discharge in patients 65 years or older (47). In their study, 14% of CCP recipients were Black/African American, 31% were Caucasian/White, 42% were Hispanic/Latinx, 34% had hypertension, 25% had diabetes, and all received 2 units of CCP. Our cohort was older, was more severely ill, and came from racial/ethnic populations at higher risk for severe COVID-19 and death (48): 26% of CCP recipients were Black/African American, 9% were White/Caucasian, 51% were Hispanic/Latinx — which was associated with mortality in our multivariable analysis — 82% had hypertension, 42% had diabetes, and all received 1 unit of CCP. This suggests social determinants of health may have adversely affected clinical outcomes of our cohort.

Reflecting practice at the peak of the pandemic at our center, the majority of patients in our cohort received corticosteroids, and corticosteroid use was associated with mortality in those requiring low-flow oxygen. In another propensity score–matched study, corticosteroid use was also associated with higher mortality (49). Among CCP recipients at least 65 years in our study, 98% received corticosteroids concurrently with or before CCP. More CCP recipients than controls also received corticosteroids in the Rogers et al. study, which did not find evidence of CCP benefit (47). In addition, although not statistically significant, a higher proportion of CCP recipients than controls received corticosteroids in the Li et al. RCT, in which there was not a signal of CCP efficacy and the median age was 70 years (25). Corticosteroid use has been associated with lower mortality in patients with COVID-19 who require mechanical ventilation (8, 9), and not in the early course of the disease (9, 50). Viral clearance was slower in patients with SARS and Middle East respiratory syndrome who received corticosteroids (51, 52), and corticosteroid use was associated with lower anti–spike protein receptor binding domain (RBD) IgG and neutralization titers in COVID-19 patients (53). Thus, we wonder if corticosteroid use further impaired the immune status of elderly patients in our cohort, who already had lower lymphocyte counts. Data from ongoing RCTs are needed to evaluate the effect of covariates, including corticosteroids, on CCP efficacy.

The ability of CCP to affect the course of COVID-19 is most likely a function of viral neutralization early in disease (14, 54). The Mayo Clinic study analysis found that high-titer CCP, as defined by signal to cutoff ratio on the OrthoV platform, reduced COVID-19 mortality relative to low-titer CCP (22). In a propensity score–matched study, compared with controls, CCP with an RBD IgG titer ≥ 1:1350 reduced mortality in nonintubated patients transfused within 72 hours of hospital admission (21). Although titers in our study cannot be directly compared with titers in other studies, CCP used in our study had high-titer spike protein IgG and a median neutralizing titer of 1:938 based, respectively, on a highly specific full-length spike protein ELISA (32, 55, 56) and a pseudovirus neutralization assay that correlates with live virus (plaque reduction) neutralization (42, 56, 57). Nonetheless, although there was a possible signal of efficacy in the subgroup younger than 65 years who received CCP within 72 hours of hospitalization, this was not the case in patients 65 years or older, who were more severely ill based on baseline data. It is possible elderly patients may require more than 1 dose of 200 mL of CCP, but 2 units did not mediate an effect in the Rogers et al. cohort (47). While there are theoretical concerns of antibody-dependent enhancement (ADE) in the presence of subneutralizing concentrations of antiviral antibodies (58, 59), ADE was not reported in other CCP treatment studies, and the levels of spike protein antibody with neutralizing capability in CCP in this study make it very unlikely. Given the high mortality of COVID-19 in patients at least 65 years and lack of evidence of CCP efficacy in this group in our study and others (25, 60), there is a need for more data on the effect of aging on CCP efficacy in COVID-19.

Consistent with other reports associating SARS-CoV-2 antibody titer with disease severity and/or mortality (61–63), spike protein IgG at enrollment was directly associated with mortality among 60 CCP recipients who had remnant sera available. Although we observed posttransfusion increases in spike protein IgG in patients younger than 65 years or who were not intubated, this cannot be distinguished from endogenous antibody without comparison with untreated controls. While we did not measure posttransfusion viral loads, pretransfusion antibody titers correlated with RT-PCR Ct values (inversely correlated with viral load). Thus, as in other studies (53, 64), endogenous antibody may have already contributed to viral control in CCP recipients. The Gharbharan et al. RCT was terminated early when it was discovered the majority of the study patients had neutralizing titers at enrollment equivalent to donor CCP (29), although the study appeared on track to meet its expected endpoint. Patients in the PLACID RCT (27) who received CCP had earlier conversion to viral RNA negativity, despite having low levels of neutralizing antibodies at enrollment. In the ConPlas-19 RCT (26), in which 49% of the patients had positive SARS-CoV-2 IgG at enrollment, CCP did not confer benefit, but there was a trend toward reduced mortality. Nonetheless, in our study as in others (27, 65), antibody titers and symptom duration associated with disease severity and a lack of evidence of CCP efficacy. This underscores the long-standing principle that convalescent antibody therapy is most likely to be effective early in the course of viral respiratory diseases (14) as shown for COVID-19 in propensity score–matched studies (23, 49) and a recently published outpatient RCT (65).

A strength of our study is that it includes patients over the age of 65 years, who represent a population with disproportionately higher COVID-19 mortality. To date, studies of CCP and other potential therapies for COVID-19 in patients negatively affected by social determinants of health are lacking. The Bronx has a higher poverty rate than the other NYC boroughs (5, 66), and our cohort was composed predominantly of Hispanic or Latinx and Black or African American populations who were severely affected by the surge conditions in NYC and experienced higher COVID-19 mortality (67–69). We attempted to control for hospital surge capacity and social determinants of health that can significantly contribute to COVID-19 outcome (70) by including week of admission, race, and ethnicity as propensity scores.

A major limitation of this study is its retrospective and nonrandomized study design. The retrospectively identified controls differed in baseline characteristics from the cases, most notably in baseline oxygen requirement, proportion of corticosteroid use, and baseline lymphocyte counts, suggesting a mismatch in severity of illness due to selection bias. While propensity score matching and multivariable analysis with adjustments were done to correct for confounding variables, there likely were additional latent and unmeasured variables that were not adjusted for. In addition, time-dependent variables such as hospital bed capacity during surge conditions and advances in clinical practice, such as use of proning techniques, lung protective ventilation strategies, and improvement in sedation, may not have been accounted for in our analysis, despite matching and adjusting for baseline week. In addition, poor or absent documentation of oxygen requirement and inconsistencies in obtaining inflammatory markers during the height of the pandemic resulted in missing data, precluding a more complete analysis. Finally, since we could not obtain antibody data for controls, we cannot assess CCP effects on antibody levels or the effect of pretransfusion antibody on CCP efficacy.

In summary, we report that CCP administration within 72 hours of hospitalization demonstrated a possible signal of reduced mortality in patients younger than 65 years. Similar to others, we found CCP was safe with no adverse events directly attributable to transfusion (21, 71, 72). Although our data suggest possible effects of age and disease severity on CCP efficacy, prospective RCTs are needed to definitively establish its efficacy. Antibody-based therapies, including CCP, have now shown promise in outpatients with mild to moderate COVID-19 (65, 73). However, nearly a year into the pandemic, effective therapies for hospitalized patients are still urgently needed, particularly for those who are elderly and at high risk for mortality. If effective in any group of hospitalized patients, CCP will have immense impact on health care resources and public health during this ongoing pandemic (74). CCP is rapidly available compared with other pharmaceuticals or vaccines and may be a more feasible option in surge conditions and/or resource-limited settings (24).

## Methods

### Patient enrollment.

One hundred three (*n* = 103) adult patients with laboratory-confirmed (nasopharyngeal PCR) COVID-19 were enrolled in the Mayo Clinic expanded access treatment protocol (31) to receive CCP between April 13 and May 4, 2020. Hospitalized patients were referred to the study team by hospitalists and/or infectious diseases consultants and were deemed eligible to receive CCP if they had been hospitalized for ≤ 3 days or were symptomatic for 3 to 7 days prior to transfusion and had severe and/or life-threatening COVID-19. Patients who were on mechanical ventilation for more than 24 hours were excluded. Severe disease was defined as respiratory symptoms with hypoxemia requiring at least 5 L of nasal cannula oxygen support. Life-threatening disease was defined as respiratory failure requiring mechanical ventilation, shock, and/or multiple organ dysfunction or failure. Ninety-one patients received CCP by day 3 of hospitalization. Patients or their legally authorized representatives provided informed consent prior to treatment.

### CCP procurement and transfusion.

After obtaining informed consent, blood was collected between March and April 2020 from otherwise healthy adult volunteers residing in Westchester County, Rockland County, and the Bronx, New York, who had recovered from COVID-19. Potential donors had a documented positive nasopharyngeal swab by PCR for SARS-CoV-2 during illness and had been asymptomatic for at least 14 days prior to sample collection. Serum was obtained by venipuncture (BD Vacutainer, serum), aliquoted, heat-inactivated at 56°C for 30 minutes, and stored at 4°C prior to antibody screening by ELISA. Donors with SARS-CoV-2 spike protein titers > 1:1000 were referred for apheresis at the New York Blood Center (NYBC). CCP from 46 MMC donors and 8 donors from the general NYBC pool was administered to patients in the study.

Plasma recipients were transfused with 1 unit (approximately 200 mL) of ABO-type matched CCP over 2–3 hours and monitored before, during, and after infusion for signs of transfusion-related reactions per standard transfusion protocol.

### Controls and data collection.

We identified 1347 non-CCP recipients with a positive SARS-CoV-2 PCR admitted to MMC between April 13 and May 4 by querying the electronic medical record (EMR). Since most CCP was administered by day 3 postadmission, baseline day was set at day +2 postadmission in non-CCP recipients. Of the 1347 identified patients, the CCP recipients and 986 non-CCP recipients were excluded because they required less than 5 L or no baseline oxygen support, had a missing baseline oxygen value (*n* = 903), were intubated for more than 24 hours at baseline (*n* = 47), or had missing data (*n* = 36). This resulted in a retrospective control group of 258 non-CCP recipients (Figure 1). We collected age, sex, BMI, race, ethnicity, comorbidities, medications, laboratory findings, and day of death or discharge from CCP-treated and control patients. Additionally, we collected duration of symptoms and hospital day of transfusion from CCP-treated patients from the EMR. Specific laboratory values and clinical characteristics were obtained from the EMR by using Structured Query Language.

For both control and CCP recipients, patient oxygen support was evaluated at day 0 and 28 post-CCP, and the corresponding day postadmission for controls, as follows: low-flow oxygen through nasal cannula or non-rebreather mask (5–15 L), high-flow nasal cannula or noninvasive ventilation, and invasive mechanical ventilation. If a patient’s oxygen requirement increased or the patient died prior to the time point of interest, the patient’s oxygenation status was considered to have worsened. Initial Ct value from the SARS-CoV-2 RT-PCR assay performed on a single platform was queried retrospectively for CCP recipients.

### SARS-CoV-2 spike protein IgG, IgM, and IgA titers before and after transfusion of CCP.

SARS-CoV-2 spike protein-binding IgG, IgM, and IgA titers were determined by ELISA using remnant sera obtained from baseline (D –1) and 1, 3, and 7 days after patients received CCP (D1, D3, and D7, respectively). Briefly, microtiter plates (Costar, Corning) were coated with 25 μL of 2 μg/mL purified spike protein (32, 55, 75) in phosphate-buffered saline (PBS) overnight at 4°C, washed with 1× PBS/0.1% Tween (PBS-T), blocked with 3% (*v/v*) milk (Bio-Rad)/PBS-T for 1 hour at room temperature (RT), washed, and incubated with heat-inactivated sera for 2 hours at RT. Plates were then washed, incubated with isotype-specific HRP-labeled goat anti-human IgG (Thermo Fisher Scientific 31410), IgM (MilliporeSigma A6907), or IgA (MilliporeSigma A0295) for 1 hour at RT. Following final washes, plates were incubated with ultra-TMB ELISA substrate (Thermo Fisher Scientific), and color development was stopped by addition of 0.5 M sulfuric acid (MilliporeSigma). Well absorbances at 450 nm (A_450_) were determined using a Cytation 5 (BioTek). The endpoint titer was determined as the highest dilution to give a signal 3 times the background A_450_ (wells with no sera).

### rVSV-SARS-CoV-2 S neutralization assay.

The neutralization assay was done as previously described (56). Briefly, CCP samples were serially diluted and incubated with pretitrated amounts of virus for 1 hour at RT; plasma-virus mixtures were added to 96-well plates (Corning) containing monolayers of Vero cells (ATCC), incubated for 7 hours at 37°C/5% CO_2_, fixed with 4% paraformaldehyde (MilliporeSigma) in PBS, washed with PBS, and stored in PBS containing Hoechst-33342 (1:2000 dilution; Invitrogen, Thermo Fisher Scientific). Viral infectivity was measured by automated enumeration of green fluorescent protein–positive cells from captured images using a Cytation 5 automated fluorescence microscope (BioTek) and analyzed using the Gen5 data analysis software (BioTek). The serum half-maximal inhibitory concentration was calculated using a nonlinear regression analysis with GraphPad Prism software.

### Study outcomes.

The primary outcome was all-cause mortality at day 28 post-CCP. The secondary outcomes were improvement in oxygenation status or mortality at day 28 post-CCP. Exploratory outcomes were associations between pre-CCP SARS-CoV-2 antibody titers and mortality at day 28.

### Statistics.

Patient characteristics and outcomes were reported as frequencies and proportions for categorical variables and median and IQR for continuous variables. Differences between groups (e.g., CCP versus non-CCP) were determined by Student’s 2-tailed *t* test or Mann Whitney *U* test for continuous variables, and χ^2^ or Fisher’s exact tests for categorical variables, as appropriate.

For the outcome analysis, we performed 1:1 propensity score matching using the nearest neighbor matching without replacement on 90 case and 258 control patients to optimize balance of baseline characteristics for assessing the independent effect of CCP on oxygenation and survival. The distribution of O_2_ requirement prior to matching showed that the cases had higher oxygen requirement (*P* < 0.001). The primary matching criteria included age, sex, race, ethnicity, BMI, week of admission, D-dimer, lymphocyte count, corticosteroid use, anticoagulation use, hypertension, diabetes, chronic pulmonary disease, chronic kidney disease, coronary artery disease, hyperlipidemia with exact matching on baseline oxygen requirement, and age group (categorical, < 65 vs. ≥ 65 years). Propensity scores were calculated using a logistic regression model. After 1:1 propensity score matching, the analysis included 73 cases and 73 controls, and the variables were not significantly different between CCP recipients and controls based on an omnibus test (*P* = 0.80) (76). The all-cause mortality at day 28 post-CCP was depicted by Kaplan-Meier curves. Differences between groups were compared using the log-rank test. Stratification analyses were done by age < 65 vs. ≥ 65 years and by baseline oxygen requirement. Corticosteroids and anticoagulation use were not well balanced in the subgroups and were further adjusted for in age-stratified analysis.

As a sensitivity analysis, factors associated with oxygenation status at day 28 were evaluated by proportional odds model, and mortality at day 28 was evaluated using logistic regression model. Adjusted OR and corresponding 95% CIs were calculated. To identify variables that predicted mortality in CCP group, we performed a stepwise model selection using the Akaike Information Criterion in a logistic regression model with age, sex, BMI, race, ethnicity, comorbidities, week of admission, time from symptom onset to transfusion, baseline oxygen requirement, anticoagulation, corticosteroid use, D-dimer, and lymphocyte count. Log-transformed SARS-CoV-2 spike protein antibody titers were individually added to select models to evaluate their association with each outcome. A 2-sided *P* value of less than 0.05 was considered statistically significant. GraphPad Prism Version 8.0, Stata/IC Version 16.1, and R were used for analysis.

### Study approval.

The retrospective cohort study, the donor plasma procurement protocol, and the use of the EAP were approved by the Albert Einstein College of Medicine Institutional Review Board. The retrospective cohort study was approved by the Albert Einstein College of Medicine Institutional Review Board for human subjects with a waiver of informed consent. All participants provided written informed consent beforehand for the donor plasma procurement protocol.

## Author contributions

HY, LP, JPD, and K Chandran designed the study. HY and LP wrote the manuscript with input from all authors. JPD, IG, K Chandran, and TW contributed to critical revision of the manuscript. HY, R Bartash, and KF enrolled patients. MP, LSR, and JU provided CCP from the blood bank. HY, UNS, K Cowman, SA, JA, AA, DB, AK, BJL, AL, ML, AM, and XAY collected clinical data. IG, RR, RT, and PS retrospectively identified control patients and collected clinical data. MRG and ASF provided clinical specimens and critical data interpretation. JR, AN, and R Babb collected clinical specimens and performed ELISAs. RHB and ASW curated clinical specimens, performed ELISAs, and analyzed ELISA data. EL, CF, DH, MED, and JMF assisted with clinical specimen processing and performed ELISAs. DH, MED, RKJ, and RHB performed and analyzed viral neutralization assays. KT, MCM, and OV performed ELISAs. GIG and RJM expressed and purified spike protein. JAQ assisted with management of clinical information. NGH, NCM, and SJG expressed, purified, and performed quality control on the spike protein. GJK and JMS assisted with specimen collection and transport. RS assisted with specimen collection. DYG provided data. TW, YL, and HY performed statistical analysis. HY, LP, JPD, K Chandran, TW, IG, UNS, DYG, and ASF assisted with data analysis and interpretation. JB, SCA, and JRL acquired funding. All authors revised and approved the final version.

## Supplementary Material

Supplemental data

## Figures and Tables

**Table 1:**
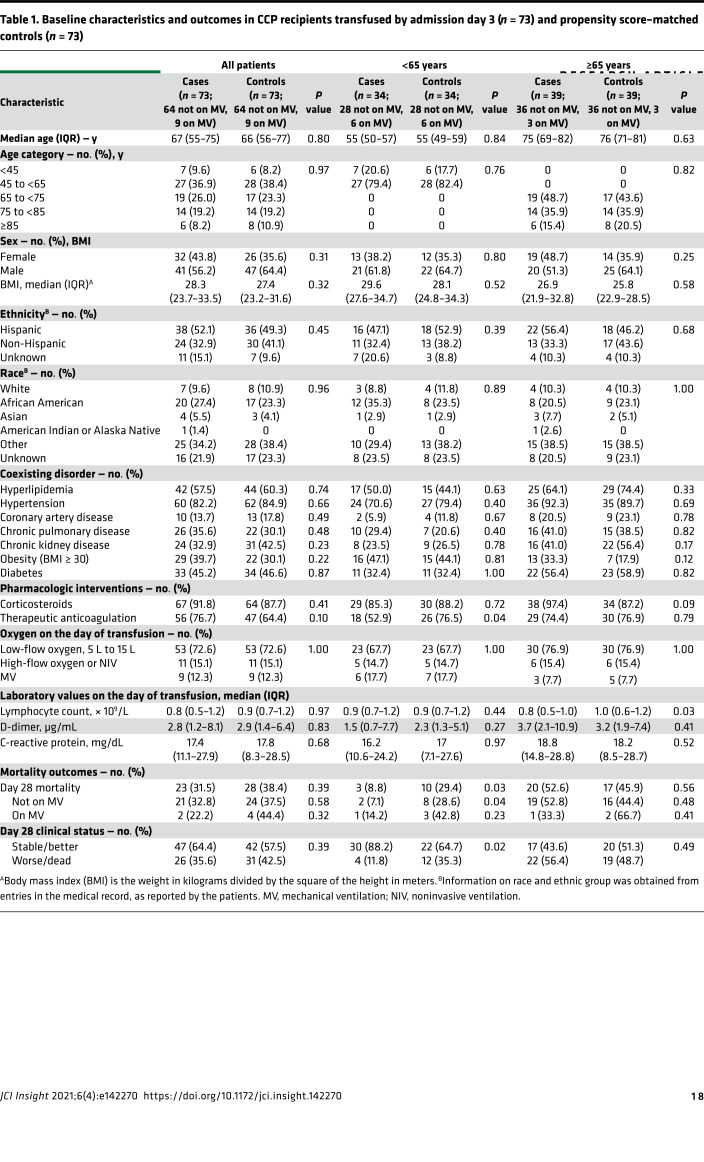
Baseline characteristics and outcomes in CCP recipients transfused by admission day 3 (*n* = 73) and propensity score–matched controls (*n* = 73)

**Table 2:**
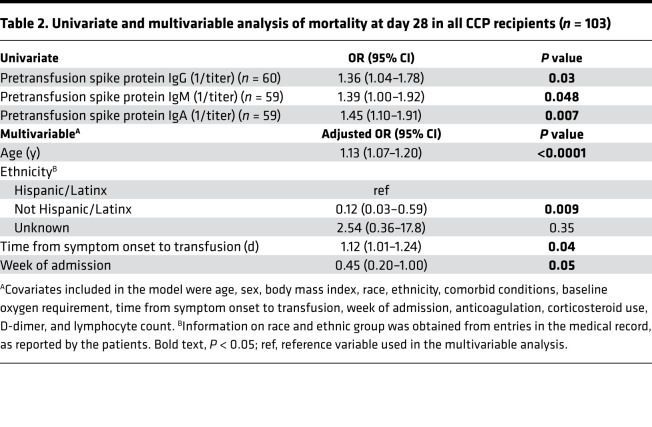
Univariate and multivariable analysis of mortality at day 28 in all CCP recipients (*n* = 103)

## Version 1 01/21/2021

In-Press Preview

## Version 2 02/22/2021

Electronic publication
