# Supplementary material for: Treatment of severe COVID-19 with convalescent plasma in Bronx, NYC
Source: JCI Insight. 2021 Feb 22;6(4):e142270. doi: 10.1172/jci.insight.142270 (PMC7934933; doi:10.1172/jci.insight.142270)
Supplement: Supplemental data [file jciinsight-6-142270-s039.pdf]

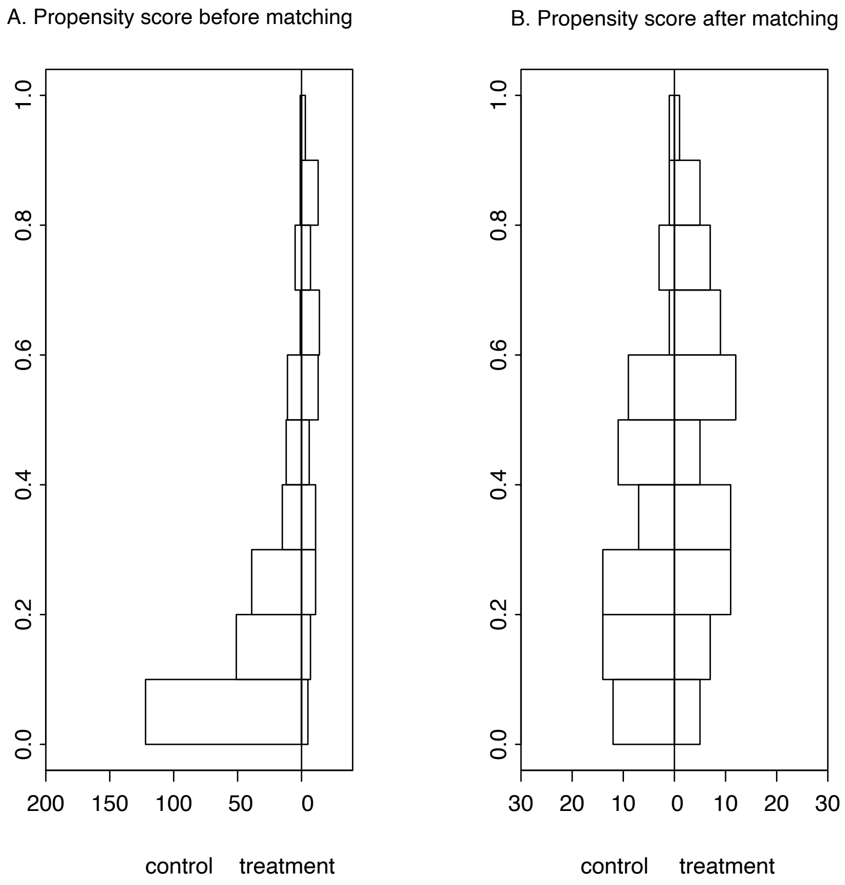

Supplemental Figure 1. Propensity scoring of CCP recipients and matched control patients. All patient comparisons before (A) and after (B) propensity score matching.

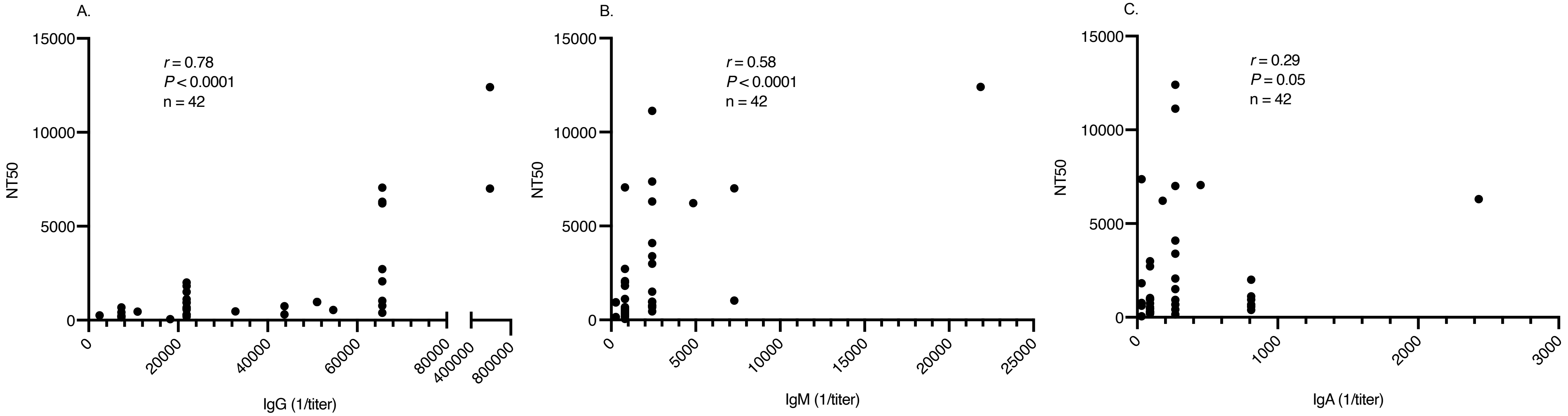

Supplemental Figure 2. Correlation between neutralizing antibody titers (NT50) and spike protein binding (A) IgG, (B) IgM and (C) IgA in CCP determined by ELISA. *r*: Spearman's correlation coefficient.

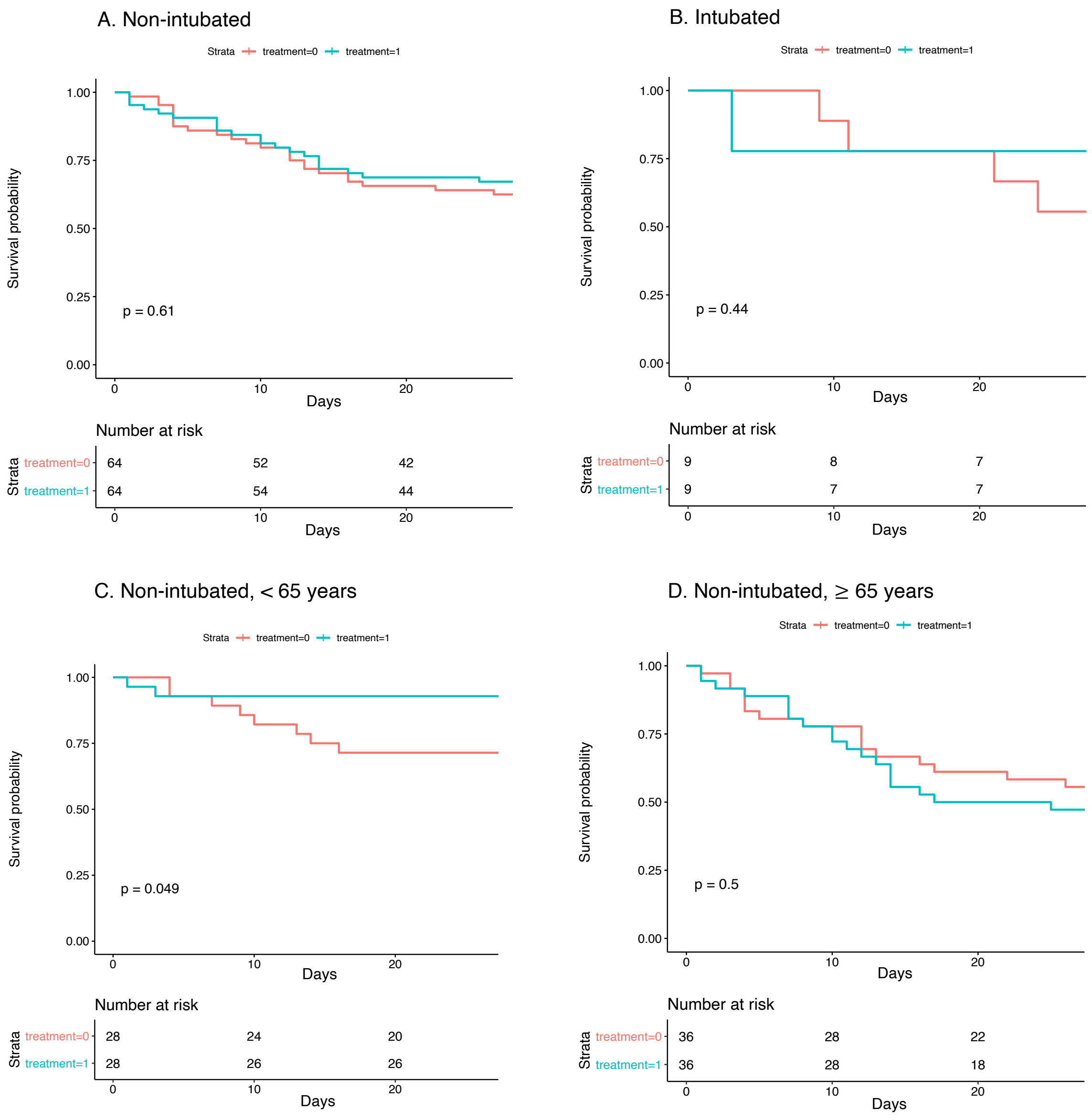

Supplemental Figure 3. Kaplan–Meier Plot of the Probability of Survival from time of transfusion to day 28 in CCP recipients (n = 73) vs matched controls (n = 73) stratified by age and intubation status at baseline. A. Non-intubated. B. Intubated. C. Non-intubated, < 65 years. D. Non-intubated, ≥ 65 years. The *P* value of a log-rank test is shown for each plot.

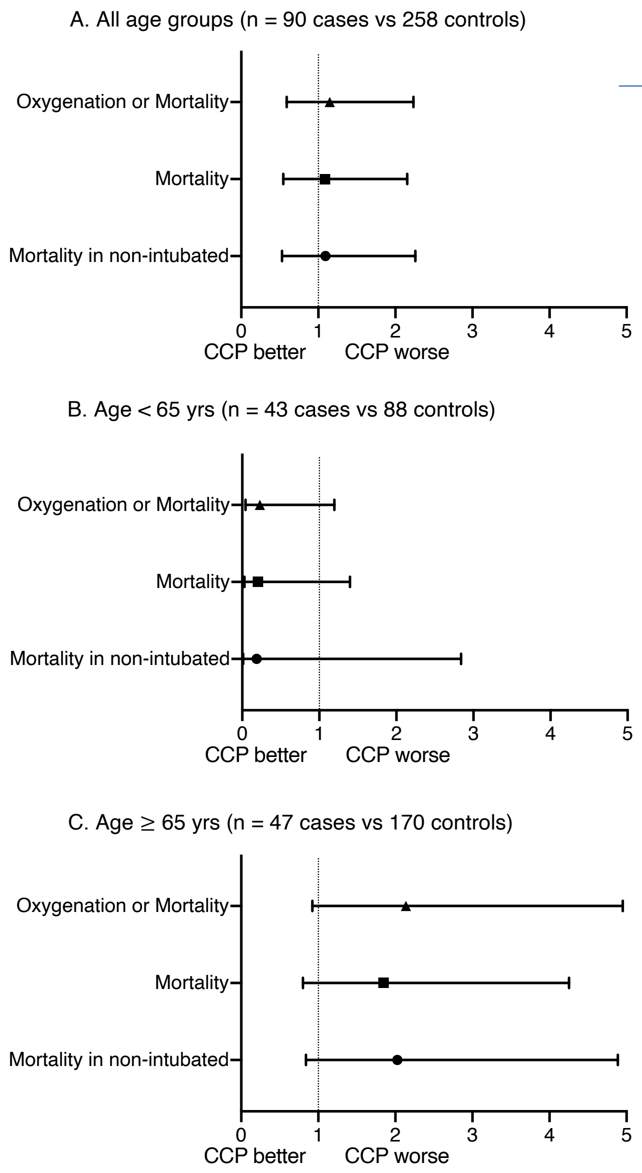

	OR	95% CI	*P* value
**A. All age groups**			
Oxygenation or Mortality	1.15	0.58 – 2.23	0.69
Mortality	1.09	0.55 – 2.15	0.82
Mortality in non-intubated	1.09	0.53 – 2.26	0.81
**B. Age < 65 yrs**			
Oxygenation or Mortality	0.23	0.04 – 1.19	0.08
Mortality	0.20	0.03 – 1.40	0.10
Mortality in non-intubated	0.19	0.01 – 2.84	0.23
**C. Age ≥ 65 yrs**			
Oxygenation or Mortality	2.14	0.92 – 4.95	0.07
Mortality	1.85	0.80 – 4.25	0.15
Mortality in non-intubated	2.03	0.84 – 4.89	0.12

Supplemental Figure 4. Day 28 outcomes in CCP recipients (n = 90) vs controls (n = 258) presented by odds ratio and 95% confidence intervals using a multivariable regression analysis. Associations were adjusted for age, sex, BMI, race, ethnicity, comorbidities, baseline oxygen requirements, week of admission, corticosteroids, therapeutic anticoagulation use, D-dimer and lymphocyte counts. A. All age groups. B. Age < 65 years. C. Age ≥ 65 years. CCP, COVID-19 convalescent plasma; CI, confidence interval; OR, odds ratio.

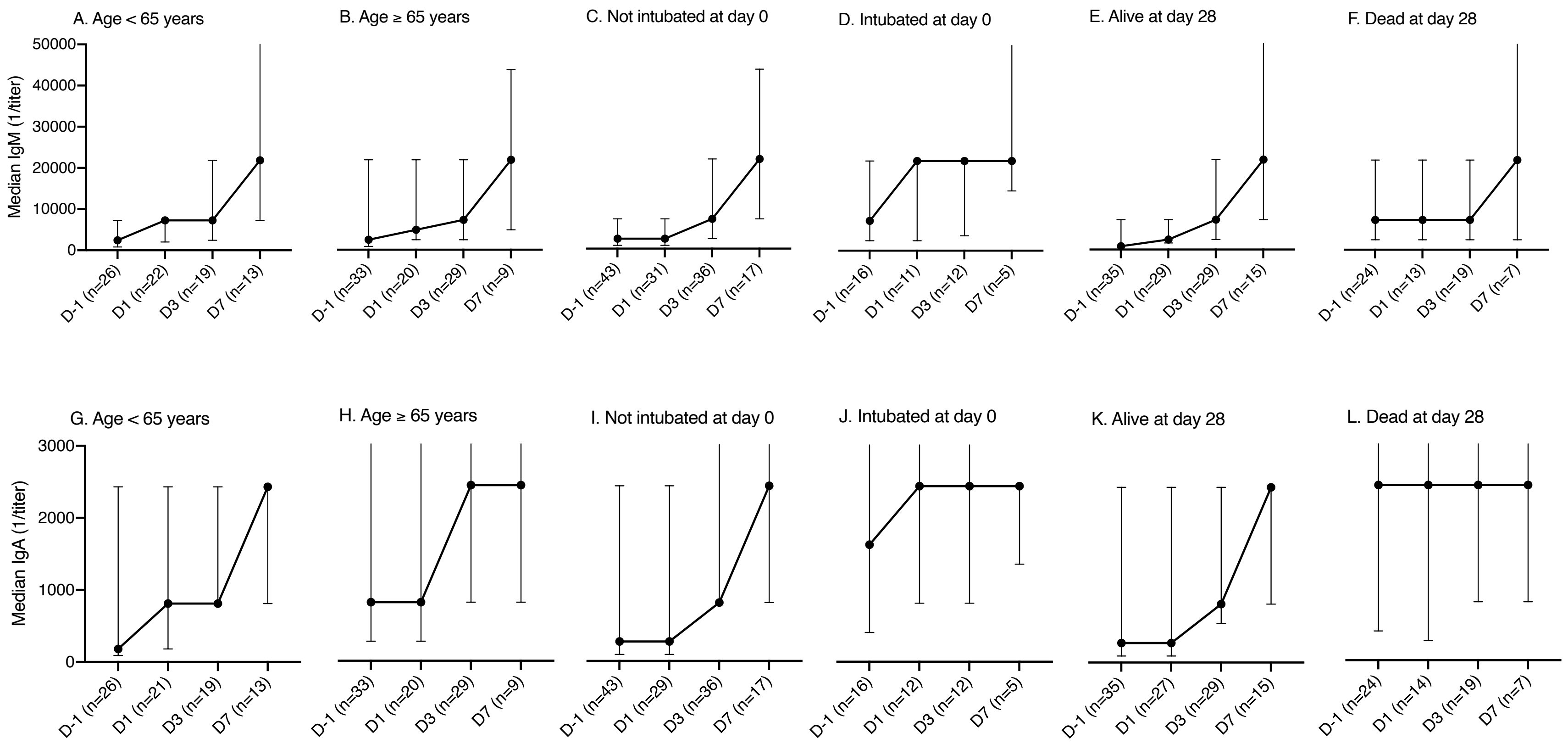

Supplementary Figure 5. SARS-CoV-2 spike protein IgM and IgA titers determined by ELISA at baseline (D-1) and 1, 3 and 7 days after transfusion (D1, D3 and D7) in CCP recipients presented by variables of interest. (A) IgM < 65 years. (B) IgM ≥ 65 years. (C) IgM patients not intubated at day 0. (D) IgM patients intubated at day 0. (E) IgM patients alive at day 28. (F) IgM patients dead at day 28. (G) IgA age < 65 years. (H) IgA age ≥ 65 years. (I) IgA patients not intubated at day 0. (J) IgA patients intubated at day 0. (K) IgA patients alive at day 28. (L) IgA patients dead at day 28. The median titers and interquartile ranges are shown on the y-axis for each time point shown on the x-axis. X-axis shows days relative to convalescent plasma transfusion.

Supplemental Table 1. Baseline characteristics and outcomes in all CCP recipients transfused by admission day 3 (n = 90) and controls before propensity score matching (n = 258).

	**All patients**			**< 65 yrs**			**≥ 65 yrs**		
**Characteristic**	**Case (n=90; 72 not on MV, 18 on MV)**	**Controls (n = 258; 243 not on MV, 15 on MV)**	***P* value**	**Case (n=43; 36 not on MV, 7 on MV)**	**Controls (n=88; 78 not on MV, 10 on MV)**	***P* value**	**Case (n=47; 36 not on MV, 11 on MV)**	**Controls (n=170; 165 not on MV, 5 on MV)**	***P* value**
Median age (IQR) - yr	66 (54-77)	72 (59-81)	**0.002**	54 (50-57)	56 (47.5-59.5)	0.17	77 (70-82)	78 (72-84)	0.10
**Age category – no. (%), yr**									
<45	9 (10.0)	17 (6.5)	0.09	9 (20.9)	17 (19.3)	0.82	0	0	
45 to < 65	34 (37.7)	71 (27.5)		34 (79.0)	71 (80.6)		0	0	
65 to <75	21 (23.3)	60 (23.2)		0	0		21 (44.6)	60 (35.2)	0.23
75 to <85	20 (22.2)	70 (27.1)		0	0		20 (42.5)	70 (41.1)	
≥85	6 (6.6)	40 (15.5)		0	0		6 (12.7)	40 (23.5)	
**Sex – no. (%), BMI**									
Female	39 (43.3)	106 (41.1)	0.71	16 (37.2)	31 (35.2)	0.82	23 (48.9)	75 (44.1)	0.55

Male	51 (56.6)	152 (58.9)		27 (62.7)	57 (64.7)		24 (51.0)	95 (55.8)	
BMI, median (IQR)[A]	28.3 (23.9-34.5)	26.7 (22.8-32.2)	**0.05**	29.3 (27.1-34.9)	30.3 (25.2-36.1)_	0.93	26.9 (22.0-32.8)	25.2 (21.9-29.2)	0.20
**Ethnicity[B]– no. (%)**									
Hispanic	46 (51.1)	113 (43.8)	0.26	21 (48.8)	42 (47.7)	0.45	25 (53.1)	71 (41.7)	0.39
Non-Hispanic	32 (35.5)	117 (45.3)		14 (32.5)	36 (40.9)		18 (38.3)	81 (47.6)	
Unknown	12 (13.3)	28 (10.8)		8 (18.6)	10 (11.3)		4 (8.5)	18 (10.5)	
**Race[B]– no. (%)**									
White	8 (8.8)	32 (12.4)	0.63	3 (6.9)	6 (6.8)	0.17	5 (10.6)	26 (15.2)	0.07
African American	23 (25.5)	69 (26.7)		16 (37.2)	20 (22.7)		7 (14.8)	49 (28.8)	
Others/Unknown	59 (65.5)	157 (60.8)		24 (55.8)	62 (70.4)		35 (74.4)	95 (55.8)	
**Coexisting disorder – no. (%)**									
Hyperlipidemia	50 (55.5)	143 (55.4)	0.98	22 (51.1)	35 (39.7)	0.21	28 (59.5)	108 (63.5)	0.62
Hypertension	74 (82.2)	222 (86.0)	0.38	30 (69.7)	65 (73.8)	0.62	44 (93.6)	157 (92.3)	0.76
Coronary artery disease	14 (15.5)	32 (12.4)	0.44	3 (6.9)	8 (9.0)	1.00	11 (23.4)	24 (14.1)	0.12

Congestive heart failure	16 (17.7)	76 (29.4)	**0.03**	4 (9.3)	15 (17.0)	0.23	12 (25.5)	61 (35.8)	0.18
Chronic pulmonary disease	30 (33.3)	89 (34.5)	0.84	13 (30.2)	22 (25.0)	0.52	17 (36.1)	67 (39.4)	0.68
Chronic kidney disease	26 (28.8)	107 (41.4)	**0.03**	9 (20.9)	25 (28.4)	0.35	17 (36.1)	82 (48.2)	0.14
Obesity (BMI ≥ 30)	35 (38.8)	80 (31.0)	0.17	20 (46.5)	45 (51.1)	0.61	15 (31.9)	35 (20.5)	0.10
Morbid obesity (BMI ≥ 35)	18 (20.0)	42 (16.2)	0.42	9 (20.9)	25 (28.4)	0.35	9 (19.1)	17 (10.0)	0.08
Diabetes	38 (42.2)	121 (46.9)	0.44	14 (32.5)	33 (37.5)	0.58	24 (51.0)	88 (51.7)	0.93
**Symptom duration**									
Median time from symptom onset to CCP, median days (IQR)	7 (5-9)	n/a		8 (5-9)	n/a		6 (4-9.5)	n/a	

**Pharmacologic interventions – no. (%)**									
Corticosteroids	84 (93.3)	163 (63.1)	**<0.0001**	38 (88.3)	55 (62.5)	**0.002**	46 (97.8)	108 (63.5)	**<0.0001**
Therapeutic anticoagulation	56 (62.2)	177 (68.6)	0.26	20 (46.5)	63 (71.5)	**0.005**	36 (76.6)	114 (67.0)	0.21
**Oxygen on the day of transfusion – no. (%)**									
Low-flow oxygen, 5L - 15L	61 (67.7)	208 (80.6)	**<0.0001**	31 (72.0)	65 (73.8)	0.68	30 (63.8)	143 (84.1)	**<0.0001**
High-flow oxygen or NIV	11 (12.2)	35 (13.5)		5 (11.6)	13 (14.7)		6 (12.7)	22 (12.9)	
MV	18 (20.0)	15 (5.8)		7 (16.2)	10 (11.3)		11 (23.4)	5 (2.9)	
**Laboratory values on the day of transfusion, median (IQR)**									
Lymphocyte count, $x10^9/L$	0.8 (0.5-1.1)	1.0 (0.8-1.4)	**0.001**	0.9 (0.7-1.2)	1.0 (0.8-1.4)	0.13	0.8 (0.5-1.0)	1.0 (0.7-1.4)	**0.001**
Not on MV	0.8 (0.6-1.1)	1.0 (0.8-1.5)	**0.001**	0.9 (0.7-1.1)	1.0 (0.8-1.5)	0.07	0.8 (0.5-1.0)	1.0 (0.7-1.4)	**0.002**
On MV	0.8 (0.5-1.6)	1.1 (0.6-1.2)	0.82	1.2 (0.6-3.9)	1.1 (0.9-1.2)	0.55	0.6 (0.5-1.6)	0.8 (0.6-1.2)	0.73
D-dimer, µg/ml	2.7 (1.1-8.3)	3.3 (1.4-7.9)	0.20	1.2 (0.6-3.2)	2.3 (1.2-5.6)	**0.01**	4.3 (2.1-11.2)	3.5 (1.7-11.0)	0.28

Not on MV	2.3 (1-5.4)	3.3 (1.4-8.5)	**0.02**	1.1 (0.6-2.7)	2.3 (1.1-6.0)	**0.003**	3.8 (2.1-9.9)	3.5 (1.7-11.0)	0.61
On MV	7.8 (2.2-14.3)	3.6 (1.5-4.7)	0.13	7.6 (1.8-9.3)	3.1 (1.5-4.7)	0.52	9.7 (2.2-17.0)	3.8 (2.0-4.2)	0.28
C-reactive protein, mg/dL	16.9 (10.3-27)	15.4 (7.7-27.2)	0.47	14.8 (8.4-22.4)	14.5 (8.1-26.6)	0.58	18.7 (14.9-28.8)	15.7 (7.6-27.4)	0.08
Not on MV	16.7 (9.5-27.1)	15.4 (7.8-27.4)	0.71	12.5 (7.3-20.1)	14.4 (8.2-26.6)	0.37	19.4 (14.8-28.9)	16 (7.7-27.6)	0.09
On MV	17.8 (14.8-24.8)	12 (6-26.2)	0.41	18.1 (11.5-28)	18.5 (6.3-26.2)	0.69	16.5 (14.9-22.9)	9.6 (4.6-12)	0.22
**Mortality Outcomes – no. (%)**									
Day 28 mortality	30 (33.3)	82 (31.7)	0.78	3 (6.9)	15 (17.0)	0.11	26 (57.7)	65 (39.8)	**0.03**
Not on MV	21 (29.1)	76 (31.2)	0.73	2 (5.5)	12 (15.3)	0.13	19 (52.7)	64 (38.7)	0.12
On MV	9 (50.0)	6 (40.0)	0.56	1 (14.2)	3 (30.0)	0.45	8 (72.7)	3 (60.0)	0.61
**Day 28 clinical status – no. (%)**									
Stable/better	57 (63.3)	172 (66.6)	0.56	39 (90.7)	70 (79.5)	0.10	17 (37.7)	97 (59.5)	**0.01**
Worse/dead	33 (36.6)	86 (33.3)		4 (9.3)	18 (20.4)		28 (62.2)	66 (40.4)	

Abbreviations: BMI, body mass index; IQR, interquartile range; MV, mechanical ventilation; NIV, noninvasive ventilation

A. Body-mass index is the weight in kilograms divided by the square of the height in meters.

B. Information on race and ethnic group was obtained from entries in the medical record, as reported by the patients. Three-category race variable was used for the logistic regression analysis given low frequency of other race categories.

Supplemental Table 2. Multivariable analysis of mortality at day 28 in CCP recipients (n = 90) vs controls (n = 258).

**Independent Variable**	**Adjusted OR (95% CI)**	***P* value**
Treatment	1.09 (0.55 – 2.15)	0.82
Age	1.06 (1.04 – 1.09)	**<0.0001**
Female Sex	0.65 (0.37 – 1.13)	0.13
Race[A]		
White	ref	
Black	0.73 (0.29 – 1.84)	0.51
Others	1.27 (0.49 – 3.29)	0.62
Ethnicity[A]		
Hispanic	ref	
Non-Hispanic	1.03 (0.46 – 2.34)	0.93
Unknown	0.77 (0.30 – 1.99)	0.59
BMI	1.00 (0.96 – 1.04)	0.97
Admission week	0.66 (0.48 – 0.92)	**0.01**
Baseline O2 requirement		
Low-flow oxygen	ref	
High-flow oxygen	1.69 (0.78 – 3.64)	0.18

Mechanical ventilation	3.29 (1.25 – 8.62)	**0.02**
Baseline D-dimer	1.10 (0.85 – 1.42)	0.46
Lymphocyte count	0.75 (0.47 – 1.21)	0.24
Corticosteroids	2.38 (1.25 – 4.50)	**0.008**
Low-flow oxygen	2.68 (1.27 – 5.68)	**0.009**
< 65 years	5.08 (0.72 – 35.8)	0.10
≥ 65 years	1.95 (0.94 – 4.04)	0.07
Therapeutic anticoagulation	0.63 (0.36 – 1.12)	0.12
Low-flow oxygen	0.56 (0.29 – 1.10)	0.09
< 65 years	1.49 (0.29 – 7.63)	0.63
≥ 65 years	0.42 (0.21 – 0.83)	**0.01**
Hypertension	0.89 (0.36 – 2.19)	0.79
Diabetes Mellitus	0.98 (0.56 – 1.73)	0.95
Chronic pulmonary disease	0.77 (0.43 – 1.37)	0.37
Chronic kidney disease	1.50 (0.84 – 2.65)	0.17
Coronary artery disease	2.56 (1.14 – 5.78)	**0.02**
Congestive heart failure	0.74 (0.38 – 1.45)	0.38
Hyperlipidemia	1.47 (0.80 – 2.70)	0.21

Abbreviations: BMI, body mass index; CI, confidence interval; OR, odds ratio

A. Information on race and ethnic group was obtained from entries in the medical record, as reported by the patients.

Supplemental Table 3. Pre-transfusion IgG, IgM and IgA titer by patient variables in CCP recipients.

Variables	IgG (1/titer), median (IQR)	*P* value	IgM (1/titer), median (IQR)	*P* value	IgA (1/titer), median (IQR)	*P* value
**Age (years)**						
< 65	18,225 (2,430-196,829), n=22	0.19	2,430 (810-2,430), n=26	0.21	180 (90-2,430), n=26	**0.04**
≥ 65	54,675 (7,290-196,829), n=38		2,430 (810-21,870), n=33		810 (270-7,290), n=33	
**Sex**						
Male	21,870 (2,430-196,829), n=31	0.70	2,430 (810-21,870), n=30	0.72	810 (90-3,645), n=30	0.69
Female	43,740 (7,290-196,829), n=29		2,430 (810-7,290), n=29		810 (90-2,430), n=29	
**Body mass index (kg/m^2)**						
< 30	43,740 (7,290-196,829), n=42	0.82	2,430 (810-21,870), n=41	0.73	810 (90-2,430), n=41	0.37

≥ 30	43,740 (4,253-196,829), n=18		2,430 (810-7,290), n=18		450 (90-2,430), n=18	
**Mechanical ventilation**						
No	21,870 (5,468-114,818), n=44	**0.009**	2,430 (810-7,290), n=43	**0.01**	270 (90-2,430), n=43	**0.02**
Yes	196,829 (65,610-196,829), n=16		7,290 (2,430-21,870), n=16		1,620 (405-6,075), n=16	
**Hospital day of transfusion**						
< 3	21,870 (7,290-196,829), n=46	0.46	2,430 (810-7,290), n=45	0.38	270 (90-2,430), n=45	0.41
≥ 3	98,415 (11,138-196,829), n=14		7,290 (810-21,870), n=14		1,620 (210-2,430), n=14	
**Days from symptom onset to transfusion**						
< 7	43,740 (7,290-196,829), n=27	0.80	2,430 (810-21,870), n=27	0.94	270 (90-2,430), n=27	0.46

≥ 7	43,740 (7,290-196-829), n=33		2,430 (810-7,290), n=32		810 (90-2,430), n=32	
**Day 28 outcome**						
Alive	14,580 (3,038-114,818), n=36	**0.02**	810 (810-7,290), n=35	**0.02**	270 (90-2,430), n=35	**0.002**
Dead	196,829 (27,338-196,829), n=24		7,290 (2,430-21,870), n=24		2,430 (405-7,290), n=24	

Abbreviations: CCP, COVID-19 convalescent plasma; IQR, interquartile range
